# Comparing the Clinical Characteristics, Laboratory Findings, and Outcomes between Epidemic and Episodic Methanol Poisoning Referrals; a Cross-sectional Study

**DOI:** 10.22037/aaem.v9i1.1278

**Published:** 2021-06-12

**Authors:** Mehdi Hadipourzadeh, Sara Ebrahimi, Pardis Ziaeefar, Nasim Zamani, Hassan Falahaty, Darren Robert, Hossein Hassanian-Moghaddam

**Affiliations:** 1 Department of Clinical Toxicology, Loghman Hakim Hospital, Shahid Beheshti University of Medical Sciences, Tehran, Iran.; 2 School of Medicine, Shahid Beheshti University of Medical Sciences, Tehran, Iran.; 3 Social Determinants of Health Research Center, Shahid Beheshti University of Medical Sciences, Tehran, Iran.; 4 Loghman Hakim Hospital, Shahid Beheshti University of Medical Sciences, Tehran, Iran.; 5 Departments of Clinical Pharmacology and Toxicology, and Renal Medicine and Transplantation, St Vincent's Hospital, Sydney, Australia.; 6 St Vincent’s Clinical School, University of New South Wales, Sydney, Australia

**Keywords:** Methanol, poisoning, disease outbreaks, renal dialysis, formaldehyde poisoning

## Abstract

**Introduction:**

Due to illegal manufacturing and sales of alcoholic beverages, epidemic outbreaks of methanol poisoning may occur. The aim of this study was to determine if there were differences in the severity, course of poisoning, and outcomes between methanol-poisoned patients admitted during an outbreak versus those who were admitted following episodic exposures.

**Methods:**

The present retrospective study was performed in a single referral poisoning center between March 2018 and March 2019 in patients with confirmed methanol poisoning. During this time, in addition to episodic cases of methanol intoxication, there were three methanol poisoning outbreaks. Outbreaks were characterized by an unexpected increase in the number of methanol-poisoned patients in a short period of time, which impacted resources and decision-making. The two groups were compared regarding their severity of poisoning, sessions of hemodialysis, and clinical outcomes.

**Results:**

Outbreak cases had a higher level of methanol than episodic cases. Odds of being dialyzed more than once was 5.4 times higher in the cases presenting during an outbreak (95% CI 2.1-14.0; p=0.001). Mean hospital stay, intubation/mechanical ventilation, and death were similar between the two groups. An evaluation of the alcoholic beverage samples available in the Iranian black market during the outbreak showed a 7-percent methanol concentration with no ethanol content.

**Conclusions:**

Poisoning risk may be higher during methanol outbreaks due to the higher methanol concentrations, requiring more hemodialysis sessions for persistent metabolic acidosis. In addition to alcohol dehydrogenase blockade, careful risk assessment of all methanol poisonings can assist with stratifying the priority for, and duration of, hemodialysis to optimize outcomes.

## 1. Introduction

Methanol is a colorless and clear liquid used in industrial solvents, antifreeze solutions, and glass cleaners. Pure methanol does not have a specific smell and it is tasteless. Worldwide, methanol poisoning is largely due to consumption of illegal homemade alcoholic beverages (1, 2). Less commonly, methanol poisoning may be the result of intentional consumption for suicidal intent. It is rapidly absorbed post-ingestion and reaches its maximum concentration within 30 to 60 minutes with a minimal lethal dose of about 1 mg/kg of body weight in adults (1). Metabolism of methanol to formic acid and formaldehyde results in toxicity and its clinical and laboratory manifestations (1).

Methanol poisoning is a medical emergency with signs/symptoms including weakness, blurred vision, nausea, vomiting, epigastric pain, headache, dyspnea, and cyanosis as well as central nervous system signs and symptoms including stupor, coma, convulsions, hypothermia, and death, particularly following a high dose of methanol and delayed treatment. Factors playing a role in the delay in receiving effective medical care are fear of punishment in countries where alcohol consumption is illegal, nonspecific signs and symptoms in the early hours of intoxication, and limited knowledge of methanol intoxication by clinicians (3). Risk factors that predict poor outcome in methanol intoxication include severe metabolic acidosis (blood pH< 7), lack of respiratory compensation, and coma (Glasgow Coma Scale (GCS) <8) on admission (4). 

If a high dose of methanol has been consumed, the mainstay of treatment is alcohol dehydrogenase (ADH) inhibition and hemodialysis (HD) to remove both methanol and its toxic metabolite, formic acid. Methanol poisoning outbreaks have occurred in Iran several times (5-7). Acute methanol intoxication continues to be an important public health concern in many Islamic countries such as Iran, Indonesia, Malaysia, Tunisia and Turkey. It is anticipated that clinical outcomes may be worse in patients presenting during an outbreak due to resources being overwhelmed (2). However, during an outbreak it is also possible that patients may present to hospital and be diagnosed and treated earlier due to increased awareness, leading to more favorable outcomes (7). This study was designed to determine if there are differences in the severity, treatment, and outcome of poisoning between methanol-poisoned patients who present during an outbreak and those who are admitted as episodic cases of this intoxication.

## 2. Methods

***2.1. Study design and setting***

This retrospective, descriptive study was conducted in a single large regional referral poisoning center during a one-year period between March 2018 and March 2019. 

The study was approved by the ethics committee in Shahid Beheshti University of Medical Sciences (Ethics committee No: IR.SBMU.RETECH.REC.1399.302). The need for informed written consent was waived because of the emergent nature of HD in the patients with signs and symptoms of severe methanol poisoning and since this was a retrospective study.

***2.2. Participants***

All patients who had consumed illicit alcoholic beverages, based on the history taken from the patient, or the relatives of critically ill patients, regardless of the presenting signs and symptoms were identified based on discharged International Classification of Disease (ICD) 10 coding and an internal database that recorded all poisoned patients who received hemodialysis. The patient’s signs and symptoms of poisoning and their venous blood gas (VBG) analysis were assessed on admission. Serum methanol concentration was measured when possible to confirm the exposure. 

The diagnosis of methanol poisoning was based on (i) a serum methanol level >6.25 mmol/L (20 mg/dL), or (ii) a clinical picture of methanol poisoning (abdominal pain, visual disturbances, dyspnea, central nervous system signs/symptoms) with both pH < 7.3 and serum bicarbonate < 20 mmol/L (8, 9). Patients with methanol poisoning were treated with oral ethanol in a fixed dosing regimen using 1 mL/Kg of five-fold diluted alcohol 96% and a maintenance dose of 0.16 mL/Kg/h based on a national guideline (1). Due to limited resources, ethanol blood concentrations could not be routinely and regularly measured in all patients.

All patients who received hemodialysis (HD) were included in this study. Indications for HD included significant metabolic acidemia (pH < 7.25 and/or bicarbonate < 15 mmol/L) with or without visual disturbances, electrolyte imbalance unresponsive to conventional therapy, and/or a serum methanol concentration > 6.25 mmol/L (20 mg/dL; the assay was intermittently available) (10). Patients who had initial normal analyses but developed signs and symptoms of poisoning and metabolic acidosis in the following hours were treated with hemodialysis. Those who died before the initiation of HD were excluded. HD was generally started between 30 minutes and eight hours after the decision was made to initiate it, depending on resource availability at the time. 

Based on an existing definition of a methanol poisoning outbreak, an unexpected increase in number of cases of methanol intoxication (a minimum number of three cases within a few days to a few weeks) (2), we categorized the patients into two groups: outbreak and episodic cases. The outbreak group consisted of patients who had presented to our unit during the methanol toxicity outbreaks (two between September-November 2018 and one in March-April 2019). Patients presenting to our unit at other times during the one-year study period were classified as episodic cases.

First, 4-hour HD was performed for every patient with a history of alcohol ingestion based on the VBG on admission or symptoms of severe intoxication including visual disturbances, seizure, and loss of consciousness. If metabolic acidosis did not resolve after the first session of HD, particularly if visual disturbances were present, a second, and even third HD session was performed. Persistence of visual disturbances without acidemia was considered a sequelae and no HD was performed in this context. 

***2.3. Data gathering***

Patients’ demographic characteristics including age, gender, amount of alcohol consumption (in mL), time elapsed between consumption and hospital presentation, clinical features on admission including signs, symptoms, Glasgow Coma Scale (GCS), and vital signs were recorded. Results of routine lab tests including serial VBG analyses, the number of HD sessions required per patients and indications for repeated HD, requirement for intubation, duration of hospital stay, and the final outcome (death, complete recovery, or recovery with neurologic/ophthalmologic sequelae) were recorded. Samples of the alcohol beverage were analyzed for the presence of methanol and ethanol on a random community sampling during outbreaks. 

***2.4. Statistical Analysis***

Data was compared between the two groups using Statistical Package for Social Sciences (SPSS) software version 21 by application of Chi-square and Mann-Whitney U test, with a significance threshold of P<0.05. Spearman rank correlation coefficient test was used to measure the strength and direction of association between two continuous variables. Enter logistic model was used to determine independent variables predicting repetition of dialysis or presentation during an outbreak in these patients. 

## 3. Results

***3.1. Baseline characteristics of studied cases***

**Figure 1:**
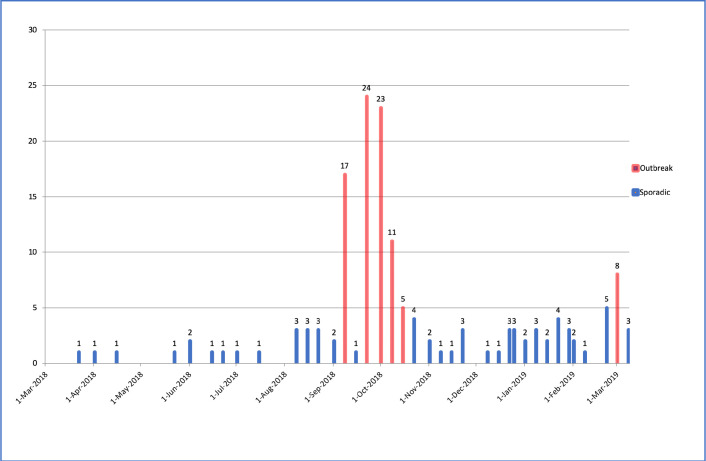
Timeline of patient referrals due to methanol poisoning in outbreak (red) or episodic (blue) events during one year in Loghman-Hakim Hospital

**Figure 2:**
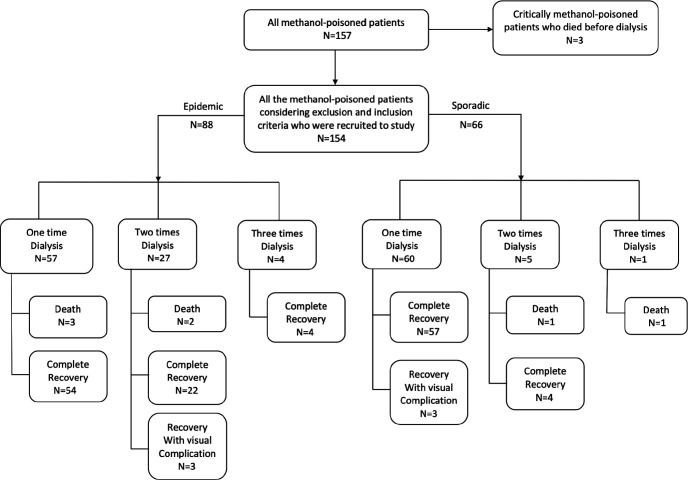
Patient recruitment chart

During the study period, 157 patients were hospitalized due to methanol poisoning. Figure 1 shows the timeline of referral cases indicating 3 outbreaks during the study period. Three patients died before initiation of HD. All other patients required HD, including twelve patients with a normal admission VBG and no noticeable symptoms, but subsequent VBG analyses during hospitalization showed metabolic acidosis so they were treated using HD. In total, 154 methanol-poisoned patients received HD and were included in the study (Figure 2; Patient inclusion chart).

The mean age was 31.9 ± 10.2 (range, 17 to 70) years. 66 of the participants were classified as episodic cases and 88 were classified as outbreak cases. There was no statistically significant difference between these two groups in terms of the amount of the alcohol consumed and presenting signs and symptoms (Table 1). However, there was a trend of delayed presentation in outbreak cases who presented after 35 hours, compared to episodic cases who had referred after 24 hours (P =0.802).

The most common signs and symptoms were blurred vision (81.8%) and gastrointestinal symptoms including vomiting (51.9%) and nausea (49. 1%). The outbreak group had a statistically higher diastolic blood pressure (P =0.005) and respiratory rate (P =0.027), and lower temperature (P =0.004) at the time of admission but the differences were not clinically significant (Table 1).

***3.2. Laboratory findings***

Laboratory testing revealed that serum methanol levels were considerably higher in the outbreak group compared to the episodic group (n=130; P = 0.011) with median (interquartile range; IQR) concentration of 11.20 mg/dL (0, 30.6) (range 0, 80.5) and 0 (0, 13.8) mg/dL (range 0, 56.5) in outbreak and episodic groups, respectively. No significant difference was detected in the admission pH, pCO_2_, and HCO_3_ of VBG analyses between the two groups. Of note, lactate dehydrogenase (LDH) was lower in outbreak group (Table 2). There was a significant correlation between LDH and admission pH (r=-0.231, p=0.011), HCO_3_ (r=-0.252, p=0.005), blood sugar (r=0.240, p=0.008), creatinine (r=0.272, p=0.002), serum potassium (r=0.297, p=0.001), and creatine kinase (r=0.205, p=0.025) on univariate analysis for the combined population.

***3.3. Outcomes***

Eight patients were intubated during hospitalization (six in the outbreak and two in the episodic group, which was a non-significant difference). More patients in the outbreak group required more than one HD treatment session (35%), compared to the episodic group (9.1%) (Table 3). Of these, 117 were dialyzed only once (57 (64.8%) in outbreak group and 60 (90.9%) in episodic group), 32 were dialyzed twice and seven patients were dialyzed three times. The indication for repeat HD was persistent isolated metabolic acidosis (n=27, 17.5%) or metabolic acidosis with visual disturbances (n=10, 6.5%). The odds of being re-dialyzed more than once was 8.2 times more in the outbreak group (95% CI 1.6-41.1; p=0.001; table 4). Despite this, hospital stay was also similar between the two groups. Final outcomes were also similar, with 91% of patients recovering completely with no sequelae, while almost 4% recovered with some neurologic or ophthalmologic complication. Death rates were similar between the groups (Table 3), and the seven deaths were related to the severity of intoxication and organ injury on presentation. 

Logistic regression analysis indicated that presenting during an outbreak, visual disturbance, and pulse rate were independent factors predicting the use of additional HD in methanol poisoned patients. Also, a higher plasma creatinine concentration and lower LDH concentration were independently associated with presenting during an outbreak (table 4). An analysis of the alcohol sample available in the black market during the outbreak showed a 7-percent pure methanol content of the beverage with no ethanol.

**Table 1:** Comparing the presenting characteristics and outcomes between epidemic and episodic methanol poisoning referrals

**Variables**	**Epidemic (n=88)**	**Episodic (n =66)**	**P **
**Gender **			
Male	76 (86.4)	57 (86.4)	0.999
**Age (years)**			
Median (IQR)	30 (24-37)	28 (25-37)	0.958
**Presenting vital signs**			
Glasgow coma scale	15 (14-15)	15 (15-15)	0.518
Systolic BP (mmHg)	110 (110-120)	110 (110-120)	0.659
Diastolic BP (mmHg)	70 (70-80)	70 (70-70)	0.005
Pulse rate (/minutes)	90 (80-100)	92 (84-100)	0.625
Respiratory rate (/minutes)	17 (14-18)	16 (14-18)	0.027
Temperature (C)	37 (36.8-37)	37 (36.9-37.4)	0.004
**Presenting chief complaint**			
Vomiting	41 (46.6)	39 (59.1)	0.126
Nausea	40 (45.5)	34 (51.5)	0.458
Abdominal pain	10(11.4)	4 (6.1)	0.259
Blurred vision	74 (84.1)	52 (78.8)	0.400
Floaters and flashes	3 (3.4)	2 (3.0)	0.896
Visual loss	6 (6.8)	6 (9.1)	0.604
Photophobia	3 (3.4)	3 (4.5)	0.719
Seizure	3 (3.4)	1 (1.5)	0.466
Headache	22 (25)	14 (21.2)	0.584
**Co-ingestion **			
Yes	5 (7.6)	2 (2.3)	0.139
**Consumption characteristic**			
Amount (cc)	500 (300-1000)	500 (300-1000)	0.915
Lag to refer to ED (hours)	35 (24-48)	24 (24-48)	0.802
**Dialysis session**			
1	57 (64.8)	60 (90.9)	0.001^†^
≥ 2	31 (35.2)	6 (9.1)	
**Hospitalization duration (days)**			
Median (IQR)	1 (1-3)	1 (1-2.5)	0.171
**Need for Intubation **			
Yes	6 (6.8)	2 (3.0)	0.295
**Final Outcome**			
Complete recovery	79 (90.8)	59 (92.2)	0.706
Recovery with complication	3 (3.4)	3 (4.7)	
Death	5 (5.8)	2 (3.1)	

Data are presented as frequency (%) or median (Inter quartile range (IQR)). BP: blood pressure, ED: emergency department. ^†^Odds Ratio 5.4 (95% CI 2.1-14.0).

**Table 2:** Comparing the laboratory findings of studied cases between epidemic and episodic methanol poisoning referrals

**Parameters**	**Epidemic (n = 88)**	**Episodic (n = 66)**	**P**
pH on presentation	7.20 (7.12-7.26)	7.21 (7.15-7.28)	0.441
PCo2 on presentation (mmHg)	28.8 (22.8-34.9)	26.6(21.6-35.7)	0.631
HCo3 on presentation (mmHg)	12 (9.3-15.7)	12 (9.5-15.8)	0.797
pH before first HD	7.20 (7.12-7.26)	7.21 (7.15-7.26)	0.737
PCo2 before first HD (mmHg)	28.8 (22.8-34.2)	26.1 (21.6-35.5)	0.414
HCo3 before first HD (mmHg)	12.0 (9.3-15.6)	12.0 (9.4-15.2)	0.762
Blood sugar (mg/dl) (n=151)	112 (94-138)	109. (93-143)	0.820
BUN (mg/dl) (n=151)	27 (22-37)	31 (24-40)	0.043
Creatinine (mg/dl) (n=151)	1.3 (1.1-1.4)	1.2 (1-1.4)	0.032
Natrium (mEq/L) (n=151)	138 (136-141)	139 (136-141)	0.320
Potassium (mEq/L) (n=152)	4.2 (4-4.4)	4.3 (4-4.6)	0.503
Methanol level (mg/dL)* (n=130)	11.2 (0-30.6)	0 (0-13.8)	0.011
Creatine phosphokinase (IU/L) (n=123)	131 (95-218)	107 (77-195)	0.306
Lactate dehydrogenase (IU/L) (n=122)	400 (331-503)	488 (400-658)	0.001
pH before second HD (n=31)	7.31 (7.29-7.39)	7.26 (7.23-7.34)	0.293
PCo2 before second HD (n=31) (mmHg)	26.5 (22.8-32.2)	27.8 (19-31.2)	0.544
HCo3 before second HD (n=31) (mmHg)	16.4 (13.2-18)	14.0 (10.2-16.7)	0.177
pH before third HD (n=7)	7.41 (7.32-7.49)	7.06 (7.06-7.06)	0.134
PCo2 before third HD (n=7) (mmHg)	26.3 (24.4-29.7)	77.9 (77.9-77.9)	0.134
HCo3 before third HD (n=7) (mmHg)	16.9 (12-21.5)	22.1 (22.1-22.1)	0.134

Data are presented as median (inter quartile range). HD: hemodialysis session. * due to long lag time between poisoning and presenting to the emergency department in some clinically confirmed cases, the serum level of methanol was undetectable. BUN: blood urea nitrogen.

**Table 3:** Comparing the outcomes of studied cases between epidemic and episodic methanol poisoning referrals

**Variables**	**Epidemic (n=88)**	**Episodic (n =66)**	**P **
**Dialysis session**			
1	57 (64.8)	60 (90.9)	0.001
≥ 2	31 (35.2)	6 (9.1)	
**Hospitalization duration (days)**			
Median (IQR)	1 (1-3)	1 (1-2.5)	0.171
**Need for Intubation **			
Yes	6 (6.8)	2 (3.0)	0.295
**Final Outcome**			
Complete recovery	79 (90.8)	59 (92.2)	0.706
Recovery with complication	3 (3.4)	3 (4.7)	
Death	5 (5.8)	2 (3.1)	

Data are presented as number (%). IQR: inter quartile range.

**Table 4:** Logistic regression analysis for independent predictive factors of repeated dialysis (dialysis sessions) and being in a methanol outbreak based on on-arrival variables

**Variable**	**Beta**	**SE **	**OR (95% CI)**	**R** ^#^	**P value**
Dialysis sessions* (1 vs. ≥2)					
Outbreak	2.10	0.82	8.16 (1.62-41.09)	0.477	<0.001
Visual disturbance	1.71	0.81	5.52 (1.13, 26.96)		
**Being in a methanol outbreak ** **(yes vs. no)†**					
Pulse rate	0.061	0.027	1.06 (1.0, 1.12)	0.379	0.012
Creatinine	2.07	1.03	7.92 (1.04, 60.21)		
Lactate dehydrogenase	-0.005	0.002	0.996 (0.995, 0.998)		

*all variables with p values less than 0.2 were entered in the model including: Creatinine, methanol level, systolic and diastolic blood pressure, temperature, pulse and respiratory rate, on-arrival pH, pCO_2_, HCO_3_, Glasgow coma scale, lag period between consumption and presentation, outbreak (yes vs. no), visual disturbance (yes vs. no).

† all variables with p values less than 0.2 were entered in the model including: Creatinine, blood urea nitrogen, lactate dehydrogenase, methanol level, diastolic blood pressure, temperature, respiratory rate, and vomiting (yes vs. no).

#: Nagelkerke R square. OR: odds ratio, SE: standard error.

## 4. Discussion

In methanol poisoning, delayed initiation or insufficient HD can contribute to complications due to this condition, including death or severe and permanent neurological injury (11). Therefore, it is advantageous if the number of required HD treatments for an individual can be predicted on admission to guide the allocation of resources and planning of treatments, especially in outbreaks when the resources are limited (8). 

According to our data, the presence of visual deficits on admission and presenting during an outbreak are independent predictors of requiring more hemodialysis treatments. A key laboratory indication for repeated HD is persistent metabolic acidosis, which was largely observed in patients presenting during an outbreak. This is interesting given that there were few statistically or clinically significant differences in admission characteristics and the time to present to hospital (although there was a non-significant trend of delayed presentation in the outbreak group). We could not confirm the exact cause of prolonged acidosis in outbreak cases, but we offer some hypotheses on the basis of these data.

One possibility is that the higher initial methanol concentration, due to intake of beverages with a high methanol content, coupled with subtherapeutic ADH blocking therapy in the context of an outbreak is associated with intermittent and prolonged metabolism of methanol to formic acid. This is a recognized risk of ethanol therapy, particularly given that blood ethanol concentrations cannot be routinely measured in our unit due to resource limitations. The risk of subtherapeutic ADH inhibition is largely removed when fomepizole is used, but unfortunately, it is too expensive for routine use in our country. 

It was interesting to note that patients needing more sessions of hemodialysis presented with significantly lower LDH concentrations, because LDH has a role in clearing endogenous acids during energy metabolism. In the case of alcohol metabolism, the NADH_2_/NAD ratio increases in both cytoplasm and mitochondria (12). Higher NADH_2_ synthesis leads to a significant reduction of pyruvate by lactate dehydrogenase. Although LDH can both reduce pyruvate and oxidize lactate, the preferred pathway is the transformation of pyruvate to lactate (13). This means that all pyruvate is reduced to lactate. Moreover, inhibition of the respiratory chain by formic acid, the most toxic metabolite of methanol, leads to a rise in the NADH_2_/NAD ratio (14). Thus, in the later stages of acute methanol poisoning, the lactate concentration may increase, which contributes to a persistent metabolic acidosis in patients with lower LDH (15). 

Regardless of the specific cellular processes involved, the significant correlation of LDH with other prognostic factors in methanol poisoning, including creatinine, serum bicarbonate, and serum blood sugar (16), prompts more research into the use of LDH for prognostication in methanol poisoning at the time of admission. 

It was interesting to note that patients who needed more sessions of hemodialysis during their hospitalization presented with statistically significant higher diastolic blood pressure and respiratory rate but a significantly lower core temperature. Lower core temperatures have previously been shown to predict poor prognosis in methanol poisoning (17). On the other hand, higher diastolic blood pressure and higher respiratory rates in patients in the outbreak group may show elevated left ventricular end diastolic pressure (18) and higher production of acid in these patients due to more severe toxicity affecting the heart and metabolic adjustment of the body, respectively. However, we did not demonstrate that the severity of poisoning was higher in these patients and, therefore, the theory of more severe acidosis causing higher respiratory rate is not confirmed here.

Interestingly, the hospitalization period did not differ significantly between the two groups although the outbreak group patients had undergone HD at least once more than the other group. The risk of complications and death were also the same between the groups, which is an encouraging finding showing that our system is managing these patients properly despite all limitations in the equipment and resources, particularly during outbreaks.

## 5. Limitations

Contrary to all the care taken to accurately record data in routine clinical practice, some data were not available in this retrospective study. Although there were some differences in trends (for example, time to present) these non-statistically significant differences between the groups may have been due to underpowering of the cohort, so future studies would be useful to validate these observations and hypotheses. 

## 6. Conclusion

During the methanol outbreaks, due to the contaminated alcoholic beverages available in the black market, poisoning can manifest with more persistent acidosis, which requires more hemodialysis sessions. Close monitoring of the patients and repeated hemodialysis until acidosis is completely resolved is advocated. Future prospective studies are warranted to further explore these clinical observations and to understand the reason for the persistent acidosis and treatment strategies that can prevent this; thus, preserving resources for methanol-poisoned patients.

## 7. List of abbreviations

ADH= Alcohol Dehydrogenase 

GCS= Glasgow Coma Scale 

HD= Hemodialysis 

ICD= International Classification of Disease

LDH= Lactate Dehydrogenase 

VBG= Venous Blood Gas

NAD= Nicotinamide adenine dinucleotide 

SPSS= Statistical Package for Social Sciences 

## 8. Declarations

### 8.1. Acknowledgements

We wish to thank Mrs. Somaye Sohrabi for data entry. This study is was based on fellowship thesis worked by Dr. Mehdi Hadipourzadeh and supported by Social Determinants Research Center, Shahid Beheshti University of Medical Sciences. 

### 8.2. Authors ‘contributions:

NZ and HHM conceived and designed the study. MH and HF acquired the data. HHM, SE and PZ checked the data separately and performed the analysis. NZ and DR drafted the manuscript. The final version of the manuscript was reviewed and approved by all authors. 

### 8.3. Funding and Support

This study was supported by a grant provided by Shahid Beheshti University of Medical Sciences (Project ID: 5050).

### 8.4. Conflict of Interest

None

### 8.5. Availability of supporting data

All collected and analyzed data can be made available by the corresponding author upon request. 
